# Larval diet and temperature alter mosquito immunity and development: using body size and developmental traits to track carry-over effects on longevity

**DOI:** 10.1186/s13071-023-06037-z

**Published:** 2023-11-22

**Authors:** Andrew J. Mackay, Jiayue Yan, Chang-Hyun Kim, Antoine M. G. Barreaux, Chris M. Stone

**Affiliations:** 1 Illinois Natural History Survey, Prairie Research Institute, University of Illinois at Urbana-Champaign, 1816 S. Oak St., Champaign, IL 61820 USA; 2 INTERTRYP (Univ. Montpellier, CIRAD, IRD), Montpellier, France; 3 School of Biological Sciences, University of Bristol, Bristol, UK

**Keywords:** Tiger mosquito, Body mass, Nutrition, Vector traits, Immune gene expression, Environmental effects

## Abstract

**Background:**

Estimating arbovirus transmission potential requires a mechanistic understanding of how environmental factors influence the expression of adult mosquito traits. While preimaginal exposure to environmental factors can have profound effects on adult traits, tracking and predicting these effects remains challenging.

**Methods:**

Using *Aedes albopictus* and a structural equation modeling approach, we explored how larval nutrition and temperature jointly affect development rate and success, female body size, and whether these metrics capture carry-over effects on adult female longevity. Additionally, we investigated how larval diet and temperature affect the baseline expression of 10 immune genes.

**Results:**

We found that larval development success was primarily determined by diet, while temperature and diet both affected development rate and female body size. Under a low larval diet, pupal wet weight and wing length both declined with increasing temperature. In contrast, responses of the two morphometric measures to rearing temperature diverged when females were provided higher larval nutrition, with pupal wet weight increasing and wing length decreasing at higher temperatures. Our analyses also revealed opposing relationships between adult female lifespan and the two morphometric measures, with wing length having a positive association with longevity and pupal weight a negative association. Larval diet indirectly affected adult longevity, and the time to pupation was negatively correlated with longevity. The expression of eight immune genes from the toll, JAK-STAT and Imd pathways was enhanced in mosquitoes with higher nutrition.

**Conclusions:**

Our results highlight deficiencies from using a single body size measure to capture carry-over effects on adult traits. Further studies of larval development rate under varying environmental conditions and its potential for tracking carry-over effects on vectorial capacity are warranted.

**Graphical Abstract:**

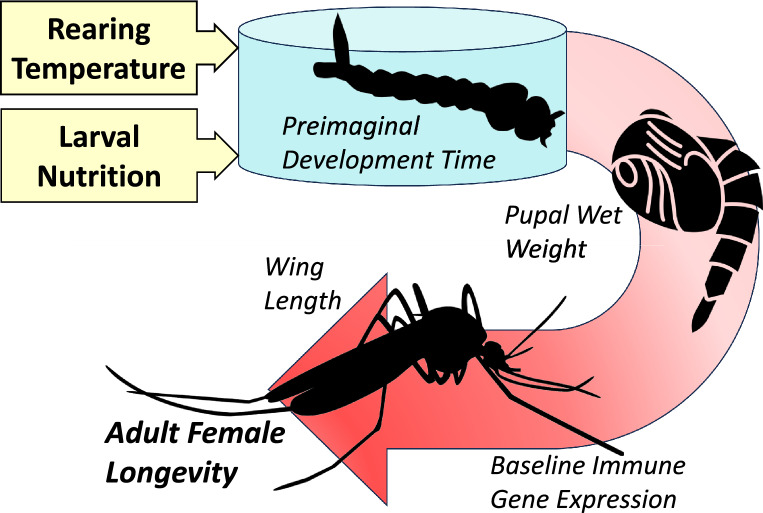

**Supplementary Information:**

The online version contains supplementary material available at 10.1186/s13071-023-06037-z.

## Background

Due to globalization and anthropogenic climate and land use change, there are increasing concerns about the spread and establishment of invasive mosquito species and their ability to transmit vector-borne diseases such as dengue or West Nile fever. To track such changing risks, vector-borne disease transmission intensity can be quantified by use of the basic reproduction number (*R*_0_) of a particular pathogen in a given environment. Focusing solely on the entomological parameters of transmission leads to the concept of vectorial capacity [1, 2]. Both vectorial capacity and *R*_0_ equations include or are composed of an incomplete set of functional traits of vectors, such as the adult survival rate, their host-biting rate, relative abundance per host, and vector competence [3]. Many of these traits are themselves composites of other traits. For instance, the vector competence of a mosquito for a given pathogen is influenced by a number of intrinsic factors, including the presence of physical barriers (e.g., midgut) within the mosquito and the interaction between different mosquito innate immune factors and that pathogen [4, 5]. Similarly, vector abundance is influenced by a range of traits, including fecundity, larval survival, and larval development rates. Although often represented as constant values in models, these traits can vary greatly under the influence of environmental factors [6]. Temperature is one such factor, and studies have shown that it affects *R*_0_ in a nonlinear manner because the underlying phenotypic traits tend to have distinct thermal optima associated with them [7, 8]. However, a range of other environmental factors influence mosquito life history traits as well, including relative humidity (RH) [9, 10], larval habitat characteristics [6, 11], and the availability and quality of larval or adult nutrition [12–15].

Predicting the effects of environmental change on mosquito population dynamics and pathogen transmission intensity is especially challenging due to the complex interrelationships among extrinsic factors that may exert influence at different stages of mosquito development. One aspect is that multiple environmental factors can jointly influence specific traits, and the magnitude and direction of the effects of these interactions can be hard to predict [10, 13, 16, 17]. Another challenge is that in some cases environmental factors may shape adult traits indirectly, as “carry-over effects” from environmental exposure in the egg, larval, or pupal (i.e., “juvenile”) life stages, or through their co-variation with certain juvenile traits [18]. For instance, the rate and amount of somatic growth mosquitoes undergo during the larval stages can subsequently affect the longevity of the adult stages [13, 19, 20]. Because such interactions can be affected by multiple variables (for instance, larval nutrition levels can be influenced by precipitation, addition of leaves or detritus, or competitive interactions within and between different species), this poses a challenge in parameterizing population dynamics and disease transmission models.

Integral projection modeling is a common approach for predicting population-level responses to environmental change based on estimates of body size and by making use of the relationships between body size and various life history traits [21–24]. If a single continuous trait, such as body size, captures the salient aspects and is a key determinant of individual fitness components, such integral projection models become a useful and appropriate approach. For mosquitoes, such an approach could potentially offer a convenient way to include the impacts of various environmental effects on adult fitness traits, as well as incorporating carry-over effects from the larval stages. It does, however, rely on the existence of a robust morphometric measure that has a predictable relationship with different life history traits. Two measures of body size are commonly used as proxies for adult fitness, wing length and pupal wet weight. While both strongly co-vary [25–27], are correlated with body mass and energetic reserves in newly emerged adults [19, 26, 28, 29], and have significant associations with key adult traits [15, 19, 30–32], allometric scaling between wing length and pupal wet weight is often nonlinear and altered by factors such as juvenile diet and temperature [33–35]. Additionally, there is increasing evidence challenging the validity of commonly held assumptions of fixed, linear relationships between body size measures and adult traits [33].

In addition to life history traits, vector competence and different aspects of mosquito immunity are likewise affected by a range of environmental factors, including temperature and adult and juvenile nutrition [36–39]. Understanding such environmental determinants of vector competence is important not only for predicting transmission under varying circumstances, but also for predicting mosquito population dynamics, if there is a correlation or trade-off between such immune responses and life history traits [40, 41]. In mosquito immune systems, the roles of immune genes from the nuclear factor kappa-light-chain-enhancer of activated B cells (NF-κB), toll, Janus kinase/signal transducers and activators of transcription (JAK-STAT), and the immune deficiency (Imd) pathways in anti-arbovirus infection have been well described [42, 43]. And as one of the antimicrobial effector molecules, antimicrobial peptides (AMPs) can be synthesized and secreted when toll and Imd pathways are activated by invading arboviruses [44]. Prior to viral exposure, however, relatively little is known of the baseline level of expression for these immune genes (i.e., baseline immune gene expression) under varying temperatures and nutritional levels.

The main objective of this study was to evaluate the effects of juvenile nutrition levels across different developmental temperatures on a number of life history traits determining vectorial capacity, specifically larval development rate and success, and adult longevity and baseline immune gene expression levels. Further, we aimed to explore whether we could identify a robust morphometric proxy for longevity by contrasting relationships with pupal wet weight and wing length.

We use *Aedes albopictus* Skuse (Diptera: Culicidae), the Asian tiger mosquito, in this study. This species, as a vector of several arboviruses including chikungunya and dengue viruses, is one of the most successful invasive species known, spreading globally from its native range in Asia, to Europe, parts of Africa, and South and North America [45]. Such success can be partly owed to its ability to develop in peridomestic containers of varying sizes that can be exposed to a range of temperatures as well as larval densities and amounts of food inputs. Therefore, an appreciation of the complex interrelationships between temperature, nutrition, and mosquito traits for this species is essential to improve our understanding of its population biology and success as an invasive mosquito, as well as the risk of increased pathogen transmission under varying environmental conditions.

## Methods

### Experimental design

Juvenile *Ae. albopictus* were reared in a 2 × 3 factorial experiment, consisting of two diet levels (1 and 2 mg of food per larva) and three rearing temperatures (20, 25, and 30 °C). The lowest temperature values are representative of the range in long-term daily average air temperatures in the Ohio River Valley region (1989–2018; NOAA [46]) during the seasonal period when *Ae. albopictus* is most active: June (22.3 °C), July (24.2 °C), August (23.4), and September (19.6 °C). The highest value is comparable to long-term daily maximum air temperatures during a period critical for population growth (July–August, 29.8 °C), and may mimic average temperatures experienced by juveniles developing in habitats directly exposed to sunlight. Previous studies have reported the average water temperature in sun-exposed, container habitats can exceed the daily mean air temperature by more than 5 °C [47, 48]. Immediately after emergence, all adults were transferred to 25 °C for assessments of immune gene expression, measurements of body size, and longevity. We expect that by maintaining adult mosquitoes in a uniform environment, external stressors (influence of temperature on energetic demands, dehydration, etc.) will be equivalent across treatments, facilitating a discrete testing of the effects of the juvenile environment on adult fitness traits. Additionally, this design may also provide a reasonable simulation of average temperatures likely to be experienced by juvenile life stages in shaded (~25 °C) and sun-exposed (~30 °C) aquatic habitats during the midsummer months, followed by convergence within shaded terrestrial microenvironments in the adult life stage (~25 °C).

### Larval rearing conditions

Mosquitoes used in this experiment were from the ninth filial (F9) generation of a colony established from field collections in Kentucky. Seventy first-instar larvae (L1) were transferred to each rearing pan with 700 ml of deionized water within 12 h of hatching. Four pans were assigned to each of the six treatment groups and reared on a 16:8 h light/dark cycle. Each pan received 10 mg (low diet) or 20 mg (high diet) of finely ground TetraMin™ Tropical Flakes (Tetra, Melle, Germany) at the start of the experiment, with the remaining food apportioned to each pan over six time intervals, as summarized in Additional file 1: Table S1. These schedules were allowed to differ between treatments to spread the additions of fresh food out over the expected development periods for different temperature and diet levels. To minimize variation in nutrient availability over the course of the experiment, and to prevent toxicity from excessive bacterial growth, the addition of the final meal to each treatment group was delayed until at least 50% of surviving individuals had reached the pupal stage.

### Body size and longevity assessment

Pupae were removed from pans daily, gently blotted to remove surface moisture, and weighed (Mettler Toledo XPR2U Ultra-Micro Balance, Columbus, OH, USA). After measuring pupal wet weight, pupae were transferred to individual screen-covered 50-ml conical centrifuge tubes containing 10 ml of deionized water. Water was removed from tubes immediately after emergence and adult females from all treatment groups were transferred to 25 °C and 81 ± 4% RH. Females were housed individually (unmated) and provided a cotton ball saturated with a 10% sucrose solution (replaced daily). Adult female survival was monitored at 24-h intervals to measure adult longevity. Wing length of all adult female specimens was measured post-mortem, from the axial incision to the wing tip, excluding the scale fringe (cellSens software ver. 1.7.1., Olympus America Inc., Waltham, MA, USA).

### Baseline immune gene expression

The baseline expression of 10 immune genes belonging to toll, JAK-STAT, and Imd pathways and their AMPs were measured in a random subset of 5-day-old adult females selected from each replicate across the period of female eclosion. Five females were chosen from each high diet replicate (20 per treatment), while only three females were collected from each low diet replicate (12 per treatment) due to the smaller number of individuals surviving to eclosion in the low diet environment. RNA extraction, copy DNA (cDNA) synthesis, and real-time polymerase chain reaction (PCR) for gene expression analysis were performed as follows: total RNA was extracted from mosquitoes (three females with low diet or five with high diet treatments from each replicate) by following the manufacturer’s protocol in NucleoSpin^®^ RNA Isolation Kit (Macherey-Nagel, Düren, Germany); a consistent cDNA transcript volume of 10 µl per reaction was synthesized by following the PrimeScript™ RT Reagent Kit with gDNA [genomic DNA] eraser (Takara Bio, Shiga, Japan), using a standard total RNA volume of 6.5 µl; real-time PCR was performed using the SensiFAST™ SYBR^®^ Hi-ROX One-Step Kit (Bioline, London, UK), and the QuantStudio™3 Real-Time PCR System (Thermo Fisher Scientific Inc., Waltham, MA, USA). The ribosomal protein S7 gene was used for normalization of cDNA templates. Relative fold changes in the expression of 10 immune genes of mosquitoes were calculated following the 2^−ΔΔCT^ method [49], using the mosquitoes reared at 25 °C as reference. All primers used for real-time PCR of the 10 immune genes are presented in Additional file 2: Table S2.

### Statistical analysis

The main and interaction effects of temperature and diet on juvenile development success (defined as survival from the L1 larval stage to complete eclosion of the adult from the pupal exuvia) were analyzed with a binomial generalized linear mixed-effects model (GLMM), with replicate included as a random effect. Attempts to similarly apply a mixed-effects model to the analysis of juvenile development time (defined as days from hatching to adult eclosion) resulted in singular fit models, due to near zero variance in the random effect (replicate) intercept; therefore, generalized linear models (GLMs) were used instead. Here, inverse transformations were applied to male and female juvenile development times, and the influences of temperature and diet and their interaction were analyzed using GLMs with a normal error distribution and an identity link function. A binomial GLM was also used to test for the influence of temperature and diet and their interaction on the sex ratio of individuals surviving to adult eclosion (i.e., proportion of newly emerged adults that were female). Main and interactive effects of diet and temperature on pupal wet weight and female wing length were examined in linear mixed-effects models (LLMs) with a normal error distribution and identity link function, and replicate as a random effect. Relationships between both female wing length and pupal wet weight were assessed using an analysis of covariance (ANCOVA). To evaluate the direct effects of the main factors (larval diet and temperature) and their interaction on adult female longevity (i.e., days surviving post-eclosion), data were initially fit to a Cox proportional hazards (PH) model [50]. To further understand how larval diet and temperature affect adult female lifespan both directly and indirectly (e.g., through their impact on larval development time and body size), data were subsequently fit to piecewise structural equation models (piecewiseSEM package; [51]). Structural equation models combine multiple variables into a causal network, where individual variables can be considered as both predictors and responses, allowing the identification of directional relationships among multiple dependent and independent variables. Here we used a multigroup comparison approach whereby longevity at each temperature was fit to individual models, with larval diet, juvenile development time, and both body size measures included as model variables. GLMs with a gamma error distribution and a log link function were used to evaluate the effects of temperature, diet, and their interaction term on relative fold change in expression of each immune gene. All analyses were performed in R (v4.2.1, R Core Team [52]).

## Results

**Fig. 1:**
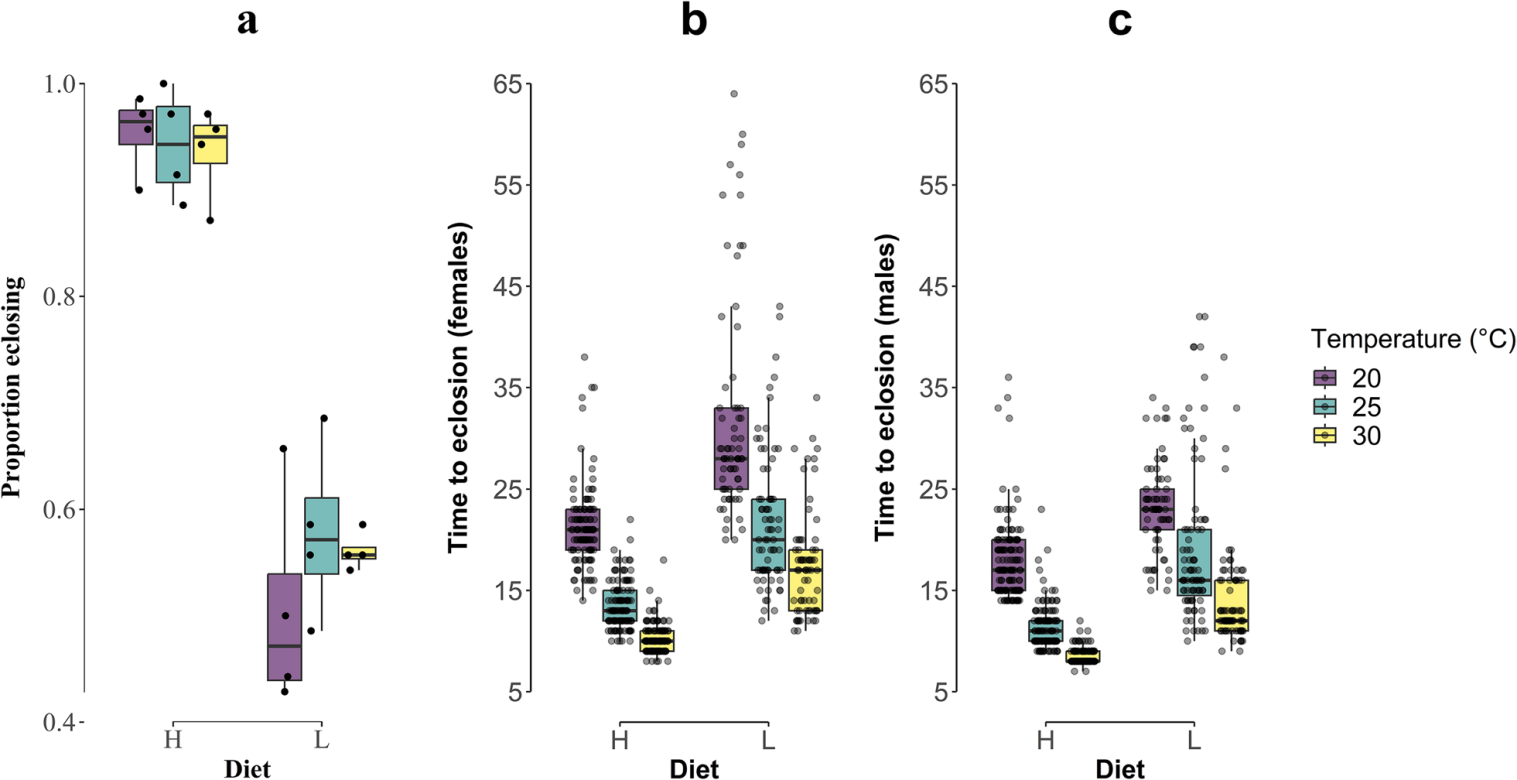
Differences in juvenile development success (**a**; proportion eclosing) and juvenile development time (L1 to adult eclosion) of female (**b**) and male (**c**) *Ae. albopictus* among temperature and diet treatment levels. **H**, high diet regime; **L**, low diet regime

There was a considerable difference in juvenile development success between the high and low larval diets (Fig. 1a). With access to the high larval diet, the proportions of L1 successfully pupated and emerged as adult mosquitoes were 0.94 ± 0.04, 0.93 ± 0.05, and 0.92 ± 0.04 (mean ± SD) at 20, 25, and 30 °C, respectively. For mosquitoes that developed under a low larval diet, the proportions that successfully emerged at 20, 25, and 30 °C were 0.49 ± 0.09, 0.56 ± 0.09, and 0.53 ± 0.02, respectively. Results of a binomial GLMM (Additional file 3: Table S3) indicated a significant positive effect of diet on juvenile survival to adulthood (*χ*^2^ = 262.9, *P* < 0.001), but no significant effect of temperature (*χ*^2^ = 1.2, *P* = 0.544) or the interaction between temperature and diet (*χ*^2^ = 2.5, *P* = 0.293). For the individuals that successfully developed to the adult stage, the mean proportion that were female was similar among treatments (range 0.45 to 0.50). A binomial GLM did not detect any significant effects of diet (*χ*^2^ < 0.1, *P* = 0.975), temperature (*χ*^2^ < 0.1, *P* = 0.973), or the interaction between temperature and diet (*χ*^2^ = 1.2, *P* = 0.542) on adult sex ratio (Additional file 4: Table S4).

In contrast to the proportion that successfully emerged, the time required to reach adulthood was dependent on both temperature and diet (Fig. 1b, c; Additional file 5: Table S5). For females, the average development times were 21.3 ± 3.9, 13.5 ± 2.32, and 10.3 ± 1.42 days under the high diet treatment, and 32.3 ± 11, 21.9 ± 6.82, and 17.5 ± 5.3 days under the low diet treatment, for 20, 25, and 30 °C, respectively. There were significant positive effects from diet (*F* = 668.0, *P* < 0.001), temperature (*F* = 633.5, *P* < 0.001), and their interaction (*F* = 42.1, *P* < 0.001), on female juvenile development time.

For males, the development times were somewhat shorter than those for females, and ranged from 17.8 ± 3.8, 11.3 ± 2.2, and 8.44 ± 0.8 days under the high diet treatment, to 23.1 ± 4.4, 19.3 ± 7.8, and 14 ± 4.8 days under the low diet treatment, at 20, 25, and 30 °C, respectively. Significant positive effects of diet (*F* = 772.3, *P* < 0.001) and temperature (*F* = 798.3, *P* < 0.001) on male juvenile development time were detected. The interaction between these factors was also significant (*F* = 61.5, *P* < 0.001), indicating that the magnitude of differences among temperatures varied by diet treatment level.

**Fig. 2:**
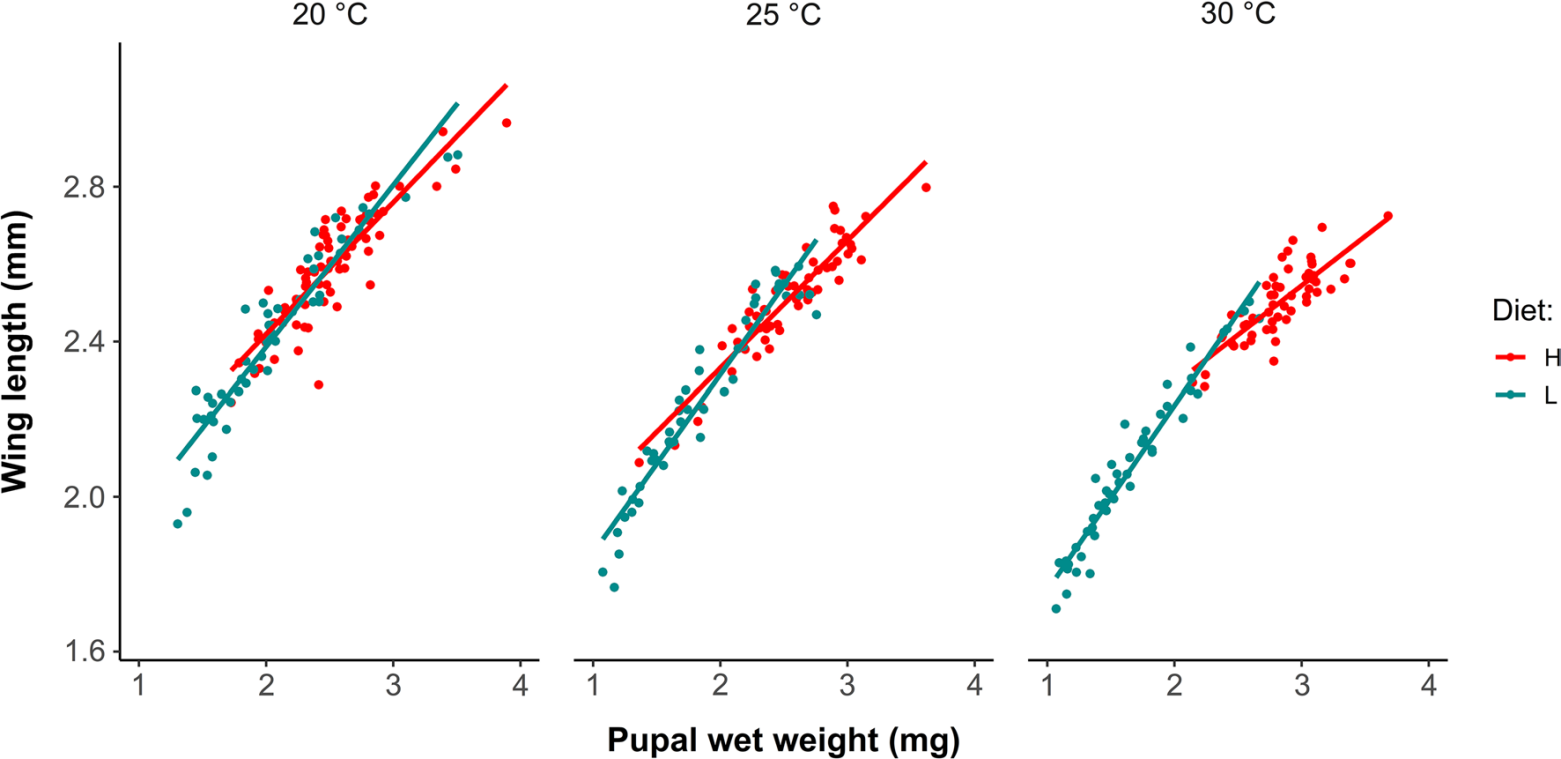
The relationship between female *Ae. albopictus* pupal wet weight and wing length shifts depending on the larval dietary regime and temperature. **H**, high diet regime; **L**, low diet regime

For female mosquitoes, we tracked their adult lifespan when given access to a carbohydrate source, and measured their wing lengths post-mortem. This allowed us both to evaluate the effects of diet and temperature on pupal wet weight and wing length, and to compare the relationship between these measures within individual females (Fig. 2; Additional file 6: Table S6). Diet had significant positive effects in LMMs of both female wing length (*χ*^2^ = 198.8, *P* < 0.001) and pupal wet weight (*χ*^2^ = 296.6, *P* < 0.001). While temperature had a significantly negative influence on female wing length (*χ*^2^ = 68.2, *P* < 0.001), the main effect of temperature was not significant in the LMM of pupal wet weight (*χ*^2^ = 4.1, *P* = 0.131). The diet–temperature interaction was significant for both wing length (*χ*^2^ = 26.1, *P* < 0.001) and pupal wet weight (*χ*^2^ = 40.9, *P* < 0.001); however, the nature of the interaction differed between the two body size measures. Female wing length declined with increasing temperature in both diet regimes, with a greater magnitude decrease under low nutrient conditions. In contrast, temperature had divergent effects on pupal wet weight between the two diet groups, with a negative influence under the low diet and a positive effect on females developing in the high diet regime (Additional file 7: Fig. S1). These differing responses to diet and temperature were reflected in the patterns of the allometric scaling observed between pupal wet weight and wing length. The slope between regression lines for pupal wet weight and wing length differed significantly for low and high diets (ANCOVA, *F* = 64.2, *P* < 0.001), but not between temperatures.

**Fig. 3:**
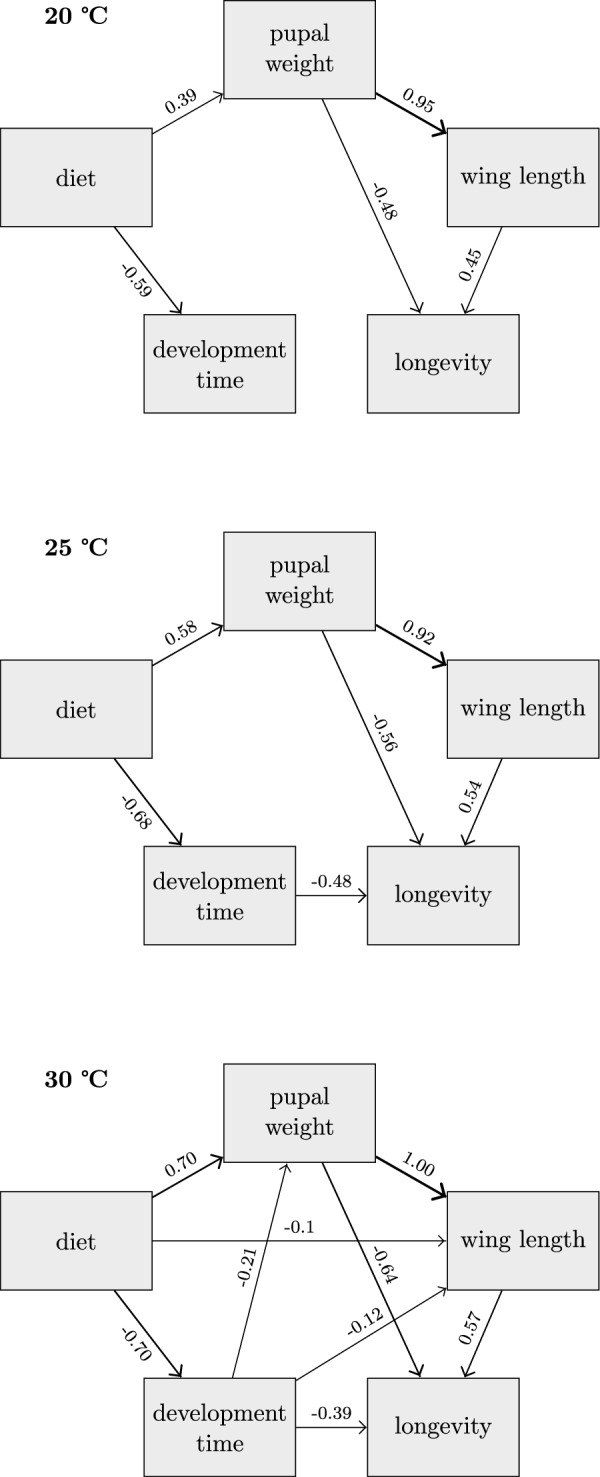
Path diagram for the multigroup partial structural equation model of adult female *Ae. albopictus* longevity. Solid lines show the significant effects and the standardized estimate associated with each, by each level of temperature experienced during larval development

Initial analyses evaluating how environmental factors during development affect adult female longevity suggested that rearing temperature did not have a significant direct influence on survival; larval diet was the only factor retained in the most parsimonious Cox PH regression model (low diet hazard ratio [HR] = 1.39; 95% confidence interval [CI] 1.13, 1.71; *P* = 0.002). Therefore, a multigroup comparison approach was employed in the subsequent piecewise structural equation model analyses to identify direct and indirect causal relationships between adult female longevity, larval diet, juvenile development time, and the two body size measures at each rearing temperature (Fig. 3). The identified model indicated that several paths were constrained among temperature levels, namely, the effect of pupal wet weight on wing length, the effect of wing length on longevity, the effect of pupal wet weight on longevity, and the effect of diet on longevity. Estimates of all other paths were allowed to vary among temperature levels. This analysis highlights that the higher larval diet level consistently decreased development time and increased pupal wet weight (*P* < 0.001 for both variables at each temperature level). Pupal weight had a significant positive effect on wing length (*P* < 0.001 at each level). Both wing length (*P* = 0.0001 at each level) and pupal weight (*P* = 0.0001 at each level) had significant, but opposing, effects on adult longevity, with wing length having a positive effect and pupal weight a negative effect. In a separate model (not shown) in which the path from pupal wet weight to adult longevity is removed, the effect of wing length on longevity becomes nonsignificant. With increasing temperature during larval development, the number of paths in the model that have a significant effect increases as well (Fig. 3). At 25 °C, the effect of larval development time on adult longevity directly becomes significant (*P* = 0.0002), with faster pupation times being associated with longer adult lifespan (Additional file 8: Fig. S2). At 30 °C, larval development time additionally shows significant negative effects on pupal wet weight (*P* = 0.003) and on adult wing length (*P* = 0.0007).

**Fig. 4:**
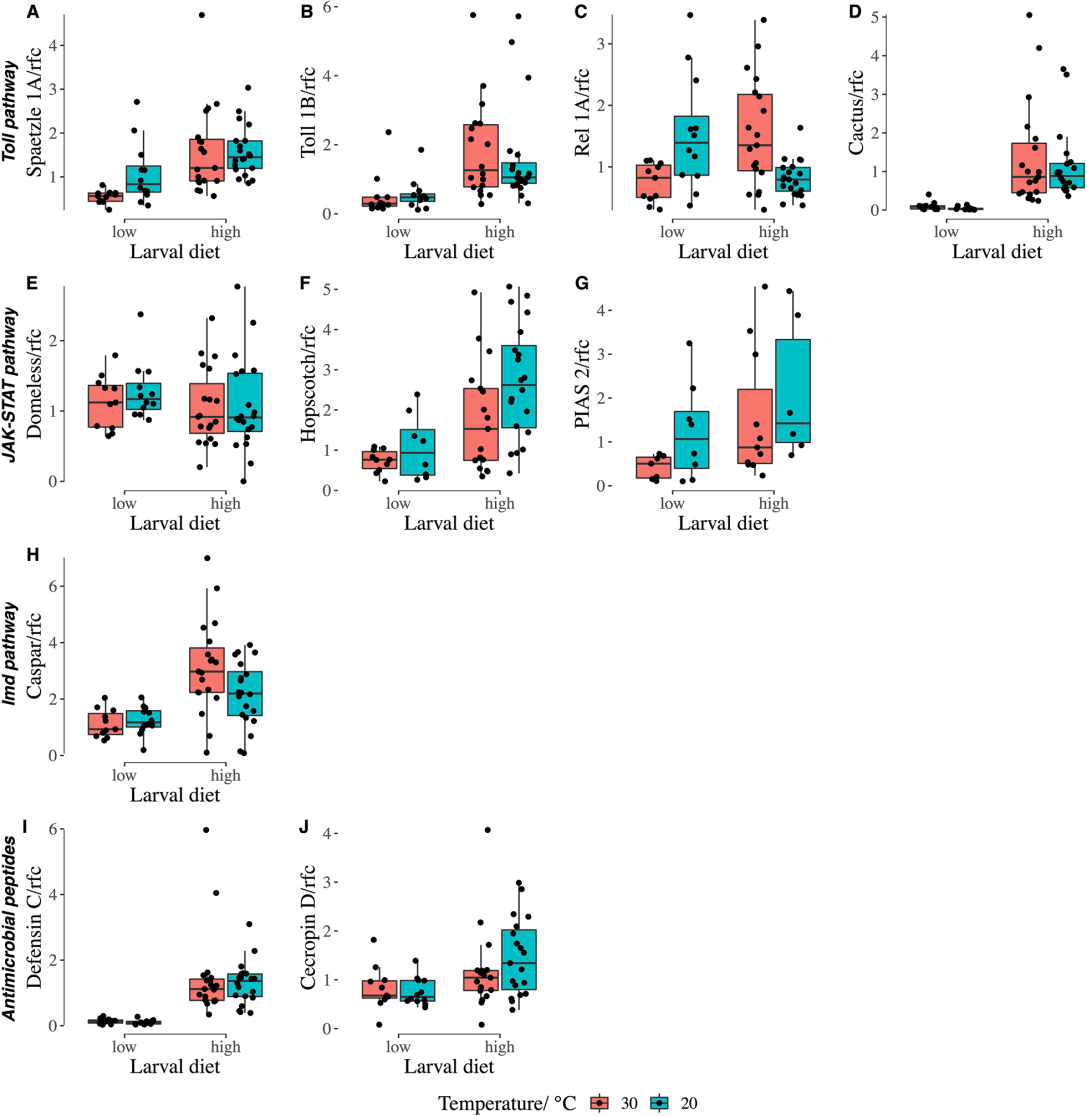
The baseline expression of 10 immune genes in 5-day-old adult female *Ae. albopictus* developing under different nutritional treatments and temperatures. Units on each *y*-axis represent the relative fold change in gene expression compared to the internal control gene S7 using the delta delta Ct method. Relative fold changes for high and low larval nutrition at 25 °C were used as the reference, respectively

Finally, we measured the baseline expression levels (i.e., without challenge of a pathogen) of several immunity-related genes to determine how these are affected by larval developmental temperature and diet (Fig. 4, Table 1). Our best-fitted GLMs showed higher baseline expression of 8 out of 10 genes belonging to the three immune pathways and AMPs at a high larval diet. In particular, upregulation of the toll pathway was found, as spaetzle (Fig. 4a), toll (Fig. 4b), and cactus (Fig. 4d) were expressed at significantly higher levels with the high larval diet than the low diet, although the expression of Rel 1A (Fig. 4c) indicated the opposite direction; a similar effect was observed in the Imd pathway, with caspar (Fig. 4h) being significantly upregulated by high larval diet; consistent with these results, the expression of defensin (Fig. 4i) and cecropin (Fig. 4j), two important AMPs regulated by the toll and Imd pathways, was also enhanced at high larval diet; the JAK-STAT pathway was also upregulated, as the expression of hopscotch (Fig. 4f) and PIAS (Fig. 4g) was significantly higher at high larval diet. Larval temperature only impacted the toll and JAK-STAT pathways, with the expression of spaetzle (Fig. 4a), Rel 1A (Fig. 4c), hopscotch (Fig. 4f), and PIAS (Fig. 4g) being significantly suppressed by higher larval development temperature (30 °C), but the expression of cactus (Fig. 4d) was enhanced in response to the high temperature. Within the toll pathway, the interaction between larval diet and temperature exhibited a stronger suppressive effect on the expression of Rel 1A (Fig. 4c, Table 1) while showing a milder effect on the expression of spaetzle (Fig. 4a, Table 1). Notably, these effects were most evident under conditions of elevated temperature.

**Table 1:** Generalized linear models of the baseline expression of immune genes in 5-day-old adult females

Predictors	Toll pathway				JAK-STAT pathway			Imd pathway	Antimicrobial peptides	
	Spa	Tol	Rel	Cac	Dom	Hop	PIA	Cas	Def	Cec
Larval diet (high)	*	***	***	***	NS	***	**	***	***	**
	0.38	1.13	−0.64	3.33	−0.12	0.91	0.95	0.80	2.44	0.51
Temperature (30 °C)	**	–	***	*	–	*	*	–	–	–
	−0.69	–	−0.73	0.93	–	−0.40	−0.69	–	–	–
Diet × temp.	*	–	***	NS	–	–	–	–	–	–
	0.69	–	1.37	−0.76	–	–	–	–	–	–

Abbreviations used in the table listed as follows. Spa: spaetzle 1A, Tol: toll 1B, Rel: Rel 1A, Cac: cactus, Def: defensin C, Cec: cecropin D, Dom: domeless., Hop: hopscotch, PIA: PIAS 2, Cas: caspar. *, ** and ***: significant level at 0.05, 0.01 and 0.001. NS: not significant. Numbers in each column of the table represent the estimates in each GLM and “–” identifies predictors that were not retained in each best fit model. Gene expression was measured by the relative fold change of Ct values compared with the internal control gene S7 and using the samples reared at 25 °C as reference samples using the delta–delta Ct method

## Discussion

We explored the impact of two environmental factors experienced by juvenile mosquitoes, temperature and diet, on larval development, the resulting mass of pupae, and the wing length of adults, and how these together affect adult longevity. Our results highlight that diet strongly affects both juvenile developmental success (i.e., survival from L1 to adult eclosion) and juvenile development time (i.e., time from hatching to adult eclosion). Additionally, juvenile development time is influenced by both the direct effect of temperature and its interaction with diet. Similarly, both these factors affect the body mass of pupae and the wing length of adult females. Larval diet was also found to alter allometric scaling between pupal wet weight and wing length, and indirectly affected adult female longevity through its effects on larval development time and body size. In addition, the baseline expression of eight genes related to the mosquito immune system was increased with access to greater nutrition during the larval stage. Understanding these indirect effects of environmental factors experienced during immature stages could improve our ability to predict transmission dynamics of arboviral diseases, and larval development time stands out as being a potentially useful proxy to track these indirect effects on longevity in *Ae. albopictus* females.

The importance of temperature and nutrient enrichment of aquatic habitats in regulating larval mosquito development and survival has been documented in previous laboratory studies and corroborated by field observations. For example, under field conditions in Puerto Rico, *Aedes aegypti* pupal productivity was found to be positively associated with the number of trees present in yards and the presence of leaf litter in containers, and negatively associated with water temperature [53]. However, while both diet and temperature are influential to the rate of *Ae. albopictus* larval development, temperatures within the range investigated in the current study (20–30 °C) have not been found to have direct effects on juvenile survivorship [20, 54–57]. Comparative studies with *Ae. aegypti* suggest modest differences in the environmental regulation of juvenile development and survivorship between these two often sympatric container species. Development success of *Ae. aegypti* juveniles appears to have greater sensitivity to temperature, relative to *Ae. albopictus* [58]. It is not clear whether this represents a difference in the biology of the study species (*Ae. aegypti* or *Ae. albopictus*) or perhaps the results of methodological differences. For example, studies assessing carry-over effects of larval nutrition frequently differ in how diet treatment levels are defined (e.g., amount per larva vs. amount per water volume), the type of diet used, and the timing of food enrichment across the developmental period. These discrepancies have the potential to affect larval development and life history traits of mosquitoes [59, 60]. In nature, the frequency of inputs and the concentration and composition of detritus accumulating in development sites are likely to be heterogeneous, and how that variability shapes development in concert with temperature is worthy of further investigation.

Mosquitoes exhibit adult body size plasticity, mediated by environmental factors experienced during development. For example, a negative association between adult body size and developmental temperatures is common among insects and other ectothermic taxa (“temperature–size rule”) [61]. Restrictions in the quantity or quality of food resources available during development can also constrain growth, resulting in a smaller adult body size [59]. Moreover, the effects of these factors on the growth of individual structures or expression of traits may be anisometric, leading to shifts in allometric relationships across different developmental environments [62]. In the current study, increasing temperature had opposing effects on the wing length (negative) and pupal wet weight (positive) of *Ae. albopictus* female mosquitoes provided adequate nutrition during development (i.e., high diet treatments), consistent with a temperature-dependent, hypoallometric relationship between adult female wing length and dry weight previously observed in this species [33]. These results showing increased body mass relative to structural size at higher developmental temperatures support the reserve-dependent growth model [63], predicting a greater allocation of dietary resources during development to energetic reserve storage in an effort to mitigate the greater metabolic demands later posed on the adult stage under a higher temperature.

The plasticity in body size in mosquitoes, responsive to larval environmental conditions, availability and quality of habitats, and intra- and interspecific competition, can lead to population-level differences in body mass between different environments [64, 65], and there is interest in understanding the consequences of this variation for population dynamics and vectorial capacity. Previous studies on the link between mosquito body size and adult longevity have in some cases suggested a positive relationship [19, 25, 66], but variable outcomes with differences between species and environmental conditions have also been reported. For instance, while a link between wing length and adult survival under conditions of low humidity was found for *Ae. aegypti*, no such effect was found for *Ae. albopictus* under similar conditions [9]. The opposite, with smaller mosquitoes living longer, or with the relationship between size and longevity being variable and dependent on environmental factors has been reported for *Anopheles gambiae* [13, 67]. Here, although we did see significant effects for both wing length and pupal weight on longevity, these effectively canceled each other out, and these measures of body size individually appeared to have little effect on longevity. Wing length and pupal weight, although strongly related to each other, do respond differently to the environmental factors we varied in this study, and the relationship between these two factors shifted as well. We could speculate that the partial structural equation model was picking up on different ways in which *Ae. albopictus* size relates to longevity, potentially through dietary restriction and lower metabolic costs improving longevity on the one hand [68], and greater structural and energetic mass improving survival on the other.

Though unable to identify congruent relationships between body size measures and longevity, we found that both a restricted larval diet and delayed maturation were negatively associated with adult lifespan in females reared at the two higher temperatures (25 and 30 °C). These results contradict earlier studies with *Ae. albopictus* and *Ae. aegypti* reporting enhanced longevity in females reared under nutrient-poor conditions [68, 69]. In both studies, it was hypothesized that dietary restriction during development would trigger changes in adult metabolism, leading to more efficient conservation of energy reserves and greater survival. Our conflicting findings may be due in part to differences in experimental design. Dietary restriction in these prior studies was achieved by reducing the rate of nutrient input without limiting the total potential resources provided to larvae (i.e., unlimited number of food additions), which allowed mosquitoes to partially compensate for a lower-quality larval environment by extending their time of development [69]. In contrast, the total food provided to larvae in our experiments was finite, negating any compensatory rewards from a long developmental period. These differences in methodology reflect the range of potential nutrient dynamics in container habitats that might result in nutrient-poor conditions—from frequent, stable inputs of resources suboptimal in their quantity or quality to more intermittent pulses of nutrient enrichment and depletion, such as from cyclical flooding or flushing of a finite resource (e.g., seasonal leaf litter accumulation in tires). Evidence from field studies suggests that the latter pattern of unstable nutrient availability is more typical of container mosquito habitats [53, 70], and these types of environments are more likely to generate resource limitations that have negative carry-over effects on adult mosquito longevity [17].

Suboptimal larval nutrition and other stressors experienced during development have been found to lead to differences in the expression of immune genes and subsequent outcomes of challenges with pathogens. However, adult body size relationships to immune gene expression and susceptibility to pathogen infection have been less consistent [71]. Smaller-bodied adult mosquitoes have been shown to be more susceptible to dengue virus infection and dissemination [72, 73], though larval nutritional stress has also been linked to a lower probability of infection by dengue virus [74] or *Plasmodium falciparum* [75] and delayed development of *P. falciparum* and therefore reduced malaria transmission capacity [76]. We evaluated the baseline expression levels of 10 immune genes in non-blood-fed and unmated *Ae. albopictus* females in the absence of an immune challenge, to determine how these could be affected by both temperature and nutrition experienced during the larval stage. Greater larval nutrition provided the most consistent and strongest effect here, significantly increasing expression for eight of the 10 immune genes that we investigated in toll (spaetzle, toll, cactus), JAK-STAT (hopscotch, PIAS), and Imd (caspar) pathways and their AMPs (defensin and cecropin), and associated with significantly reduced expression of only one immune gene (Rel 1A). Mounting an immune response in insects costs energy and consumes proteins to provide essential amino acids for the synthesis of components and effectors of immune pathways [77, 78], such as AMPs [79]. Here, larval diet provides energy and the only protein source for our studied mosquitoes, and therefore significantly modulates their immune performance [80]. This is in agreement with a previous study without a viral challenge, where an association between a higher-protein diet for larval *Drosophila melanogaster* and an increased constitutive transcription of two genes encoding defensive AMPs was found [81]. In a more recent study with a viral challenge, a similar effect of larval nutrition on immune gene expression of *Ae. aegypti* was found, with the expression for 9 out of 14 genes from the same immune pathways being suppressed in individuals that experienced nutritional stress during the larval stage, compared with their counterparts reared at higher larval nutrition [73]. Although Telang et al. [38] found greater basal expression of spaetzle, cecropin and defensin in *Ae. aegypti* derived from malnourished larvae, methodological differences including the studied species, larval diet composition, and the timing of gene expression measurement may account for the contradictory findings. High temperature had a negative impact on the expression of four genes belonging to the toll (spaetzle and Rel 1A) and JAK-STAT (hopscotch and PIAS) pathways in this study, corroborating previous work showing that this environmental factor could drastically affect immune responses [37]. The interaction between larval diet and temperature significantly affected only two genes (spaetzle and Rel 1A). It is possible that more pronounced effects, and interactions, could be evident when testing immune responses in the face of immune challenge.

## Conclusions

This work adds to a growing body of work that demonstrates the complex interactions between environmental factors that influence larval development and potentially carry over to the adult stage. Since such carry-over effects—particularly if they affect traits such as longevity or within-vector pathogen dynamics—would affect transmission capacity, there is a strong interest in being able to track and include such effects in predictive models. As such, we wanted to see whether a useful morphometric or developmental proxy could be identified. While wing length and pupal wet weight did not seem to be convincing morphometric proxies for adult longevity in this study, we did find evidence of a relationship with the time it took for larvae to develop to the pupal stage, at least under the medium- and high-temperature levels. This raises certain questions for further research, such as the specific mechanisms underlying this association between larval development rate and longevity, and whether this relationship would hold under more natural conditions with variable temperatures and influxes of detritus and across a range of larval densities. Finally, there would have to be a practical way to measure this rate under field conditions. Given, however, that this trait is already included in many population dynamics models, it would make an appealing proxy for carry-over effects on longevity, and is worthy of further research.

### Supplementary Information

**Additional file 1. Table S1**: Days post-hatch when 10 mg (low diet) or 20 mg (high diet) of finely ground TetraMin™ was added to all replicate larval rearing pans in each treatment group. The seventh meal was added when the cumulative number of pupae collected in each treatment group exceeded the number of remaining live larvae.

**Additional file 2. Table S2**: The primers used for real-time PCR.

**Additional file 3. Table S3**: Generalized linear mixed model of the influence of larval diet and rearing temperature on *Ae. albopictus* juvenile development success (survival L1 to adult eclosion).

**Additional file 4. Table S4**: Generalized linear model of the influence of larval diet and rearing temperature on the sex ratio of *Ae. albopictus* surviving to adult eclosion.

**Additional file 5. Table S5**: Generalized linear models of the influence of larval diet and rearing temperature on inverse transformed juvenile development time (1/days from L1 to adult eclosion) of male and female *Ae. albopictus*.

**Additional file 6. Table S6**: Linear mixed models of the influence of larval diet and rearing temperature on female *Ae. albopictus* body size.

**Additional file 7. Figure S1**: Effects of temperature and diet (H, high; L, low) on female *Ae. albopictus* wing length and pupal wet weight. Mean values of groups sharing the same letter were not significantly different (LMM post hoc Tukey pairwise comparisons, *P* ≥ 0.05). H, high diet regime; L, low diet regime.

**Additional file 8. Figure S2**: The relationship between larval development time (L1 to pupation) and adult female longevity for the high and low diets under three different temperatures experienced during the larval stage. H, high diet regime; L, low diet regime.

## Data Availability

The data described in this article can be freely and openly accessed from the Dryad digital repository: 10.5061/dryad.70rxwdc2t.

## Abbreviations

*Ae.*: *Aedes*
AMP: Antimicrobial peptide
Cac: Cactus gene
Cas: Caspar gene
cDNA: Copy DNA
Cec: Cecropin D gene
Def: Defensin C gene
Dom: Domeless gene
Hop: Hopscotch gene
L1: First-instar larva
PIA: PIAS2 gene
*Rel*: Rel 1A gene
RH: Relative humidity
*Spa*: Spaetzle 1A gene
*Tol*: Toll 1B gene
