# Supplementary material for: Larval diet and temperature alter mosquito immunity and development: using body size and developmental traits to track carry-over effects on longevity
Source: Parasit Vectors. 2023 Nov 22;16:434. doi: 10.1186/s13071-023-06037-z (PMC10666368; doi:10.1186/s13071-023-06037-z)
Supplement: Supplementary file 1 — Additional file 1. Table S1: Days post-hatch when 10 mg (low diet) or 20 mg (high diet) of finely ground TetraMin™ was added to all replicate larval rearing pans in each treatment group. The seventh meal was added when the cumulative number of pupae collected in each treatment group exceeded the number of remaining live larvae. [file 13071_2023_6037_MOESM1_ESM.docx]

**Table S1.** Days post-hatch when 10 mg (low diet) or 20 mg (high diet) of finely-ground TetraMin^TM^ was added to all replicate larval rearing pans in each treatment group. The 7^th^ meal was added when the cumulative number of pupae collected in each treatment group exceeded the number of remaining live larvae.

| **Temperature (°C)** | **Diet**  **Level** | **Sequential meal** | | | | | | |
| --- | --- | --- | --- | --- | --- | --- | --- | --- |
|  |  | **1st** | **2nd** | **3rd** | **4th** | **5th** | **6th** | **7th** |
| 20 | low | 0 | 3 | 6 | 9 | 12 | 16 | 21 |
|  | high | 0 | 3 | 6 | 9 | 11 | 13 | 16 |
| 25 | low | 0 | 3 | 5 | 7 | 9 | 11 | 13 |
|  | high | 0 | 2 | 4 | 6 | 7 | 8 | 9 |
| 30 | low | 0 | 2 | 4 | 6 | 7 | 8 | 11 |
|  | high | 0 | 2 | 3 | 4 | 5 | 6 | 7 |
