# Supplementary material for: Larval diet and temperature alter mosquito immunity and development: using body size and developmental traits to track carry-over effects on longevity
Source: Parasit Vectors. 2023 Nov 22;16:434. doi: 10.1186/s13071-023-06037-z (PMC10666368; doi:10.1186/s13071-023-06037-z)
Supplement: Supplementary file 2 — Additional file 2. Table S2: The primers used for real-time PCR. [file 13071_2023_6037_MOESM2_ESM.docx]

**Table S2.** The primers used for real-time PCR

| **Transcript ID** |  | **Primers’ name** |  | **Primer sequence (5’ to 3’)** |  | **Reference** | | |
| --- | --- | --- | --- | --- | --- | --- | --- | --- |
| AAEL007696-RA |  | Rel1A Forward |  | TGGTGGTGGTGTCCTGCGTAAC |  | | Xi et al. 2008 |  |
|  |  | Rel1A Reverse |  | CTGCCTGGCGTGACCGTATCC |  | |  |  |
| AAEL003507-RA |  | Toll1B Forward |  | TCTCATCAGGATTCCACAAC |  | | Shin et al. 2006 |  |
|  |  | Toll1B Reverse |  | CGGTTGAAATTCCGACGAAG |  | |  |  |
| AAEL013434-RA |  | Spaetzle1A Forward |  | GACAAAGAACGGATGCCAAT |  | | Pan et al. 2012 |  |
|  |  | Spaetzle1A Reverse |  | TTCGAGGGAATCATTTGGAC |  | |  |  |
| AAEL003832-RA |  | DefensinC Forward |  | TGTTCGCTCTTACGCCAACT |  | | Xi et al. 2008 |  |
|  |  | DefensinC Reverse |  | ATCTCCTACACCGAACCCACT |  | |  |  |
| AAEL000709-RA |  | Cactus Forward |  | AGACAGCCGCACCTTCGATTCC |  | | Xi et al. 2008 |  |
|  |  | Cactus Reverse |  | CGCTTCGGTAGCCTCGTGGATC |  | |  |  |
| AAEL000598-RA |  | CecropinD Forward |  | ATGAACTTCACTAAGCTGTT |  | | Pan et al. 2012 |  |
|  |  | CecropinD Reverse |  | TCATTTTCCAATCGCTTTTAT |  | |  |  |
| AAEL003579-RA |  | Caspar Forward |  | GAATCCGAGCGAGCCGATGC |  | | Xi et al. 2008 |  |
|  |  | Caspar Reverse |  | CGTAGTCCAGCGTTGTGAGGTC |  | |  |  |
| AAEL012471-RA |  | Domeless Forward |  | AAACGGTGGCAAAATGAACT |  | | Souza-Neto et al. 2009 |  |
|  |  | Domeless Reverse |  | CATACAGCCGGCTTTCTTCT |  | |  |  |
| AAEL012553-RA |  | Hopscotch Forward |  | ACAGGCACAGGCCGAAAA |  | | Carvalho-Leandro et al. 2012 |  |
|  |  | Hopscotch Reverse |  | CCGTTGGACAGCTCGATAAAG |  | |  |  |
| LOC5579515 |  | PIAS2 Forward |  | GCTGCAACGCATGAAAACTA |  | | Souza-Neto et al. 2009 |  |
|  |  | PIAS2 Reverse |  | CAGACGGGACAGTTCCAAGT |  | |  |  |
| AAEL009496-RA |  | S7 Forward |  | GGGACAAATCGGCCAGGCTATC |  | | Xi et al. 2008 |  |
|  |  | S7 Reverse |  | TCGTGGACGCTTCTGCTTGTTG |  | |  |  |

**Table S2 references.**

Carvalho-Leandro, D., Ayres, C., Guedes, D., Suesdek, L., Melo-Santos, M., Oliveira, C., Cordeiro, M., Regis, L., Marques, E., Gil, L., & Magalhaes, T. (2012) Immune transcript variations among Aedes aegypti populations with distinct susceptibility to dengue virus serotype 2. *Acta tropica,* **124**, 113-119. <https://doi.org/10.1016/j.actatropica.2012.07.006>

Pan, X., Zhou, G., Wu, J., Bian, G., Lu, P., Raikhel, A., & Xi, Z. (2012) Wolbachia induces reactive oxygen species (ROS)-dependent activation of the Toll pathway to control dengue virus in the mosquito Aedes aegypti. *Proceedings of the national academy of sciences,* **109**, E23-E31. <https://doi.org/10.1073/pnas.1116932108>

Shin, S., Bian, G., & Raikhel, A. (2006) A toll receptor and a cytokine, Toll5A and Spz1C, are involved in toll antifungal immune signaling in the mosquito Aedes aegypti. *Journal of Biological Chemistry,* **281**, 39388-39395. <https://doi.org/10.1074/jbc.M608912200>

Souza-Neto, J., Sim, S., & Dimopoulos, G. (2009) An evolutionary conserved function of the JAK-STAT pathway in anti-dengue defense. *Proceedings of the national academy of sciences,* **106**, 17841-17846. <https://doi.org/10.1073/pnas.0905006106>

Xi, Z., Ramirez, J., & Dimopoulos, G. (2008) The Aedes aegypti toll pathway controls dengue virus infection. *PLoS pathogens,* **4**, e1000098. <https://doi.org/10.1371/journal.ppat.1000098>
