# Supplementary material for: Larval diet and temperature alter mosquito immunity and development: using body size and developmental traits to track carry-over effects on longevity
Source: Parasit Vectors. 2023 Nov 22;16:434. doi: 10.1186/s13071-023-06037-z (PMC10666368; doi:10.1186/s13071-023-06037-z)
Supplement: Supplementary file 3 — Additional file 3. Table S3: Generalized linear mixed model of the influence of larval diet and rearing temperature on Ae. albopictus juvenile development success (survival L1 to adult eclosion). [file 13071_2023_6037_MOESM3_ESM.docx]

**Table S3.** Generalized linear mixed model of the influence of larval diet and rearing temperature on *Ae. albopictus* juvenile development success (survival L1 to adult eclosion).

| **Effect** | **Estimate ± SE** | ***z* value** | **Pr > *z*** |
| --- | --- | --- | --- |
| Intercept | 2.798 ± 0.260 | 10.8 | <0.001 |
| Temperature (25 °C) | -0.283 ± 0.343 | -0.8 | NS |
| Temperature (30 °C) | -0.334 ± 0.340 | -1.0 | NS |
| Diet (low) | -2.841 ± 0.284 | -10.0 | <0.001 |
| Temperature (25 °C) x Diet (low) | 0.557 ± 0.383 | 1.5 | NS |
| Temperature (30 °C) x Diet (low) | 0.492 ± 0.380 | 1.3 | NS |
