# Supplementary material for: Larval diet and temperature alter mosquito immunity and development: using body size and developmental traits to track carry-over effects on longevity
Source: Parasit Vectors. 2023 Nov 22;16:434. doi: 10.1186/s13071-023-06037-z (PMC10666368; doi:10.1186/s13071-023-06037-z)
Supplement: Supplementary file 4 — Additional file 4. Table S4: Generalized linear model of the influence of larval diet and rearing temperature on the sex ratio of Ae. albopictus surviving to adult eclosion. [file 13071_2023_6037_MOESM4_ESM.docx]

**Table S4.** Generalized linear model of the influence of larval diet and rearing temperature on the sex ratio of *Ae. albopictus* surviving to adult eclosion.

| **Effect** | **Estimate ± SE** | ***z* value** | **Pr > *z*** |
| --- | --- | --- | --- |
| Intercept | -0.168 ± 0.124 | -1.4 | 0.175 |
| Temperature (25 °C) | 0.052 ± 0.176 | 0.3 | 0.766 |
| Temperature (30 °C) | 0.137 ± 0.176 | 0.8 | 0.435 |
| Diet (low) | 0.168 ± 0.212 | 0.8 | 0.426 |
| Temperature (25 °C) x Diet (low) | -0.181 ± 0.293 | -0.6 | 0.538 |
| Temperature (30 °C) x Diet (low) | -0.327 ± 0.296 | -1.1 | 0.269 |
