# Supplementary material for: Larval diet and temperature alter mosquito immunity and development: using body size and developmental traits to track carry-over effects on longevity
Source: Parasit Vectors. 2023 Nov 22;16:434. doi: 10.1186/s13071-023-06037-z (PMC10666368; doi:10.1186/s13071-023-06037-z)
Supplement: Supplementary file 5 — Additional file 5. Table S5: Generalized linear models of the influence of larval diet and rearing temperature on inverse transformed juvenile development time (1/days from L1 to adult eclosion) of male and female Ae. albopictus. [file 13071_2023_6037_MOESM5_ESM.docx]

**Table S5.** Generalized linear models of the influence of larval diet and rearing temperature on inverse transformed juvenile development time (1 / days from L1 to adult eclosion) of male and female *Ae. albopictus*.

| **Sex** | **Effect** | **Estimate ± SE** | ***t* value** | **Pr > *z*** |
| --- | --- | --- | --- | --- |
| Female | Intercept | 0.048 ± 0.001 | 45.3 | <0.001 |
|  | Temperature (25 °C) | 0.028 ± 0.002 | 18.4 | <0.001 |
|  | Temperature (30 °C) | 0.051 ± 0.001 | 34.0 | <0.001 |
|  | Diet (low) | -0.015 ± 0.002 | -8.2 | <0.001 |
|  | Temperature (25 °C) x Diet (low) | -0.012 ± 0.002 | -4.8 | <0.001 |
|  | Temperature (30 °C) x Diet (low) | -0.023 ± 0.003 | -9.2 | <0.001 |
| Male | Intercept | 0.058 ± 0.001 | 52.8 | <0.001 |
|  | Temperature (25 °C) | 0.033 ± 0.002 | 21.1 | <0.001 |
|  | Temperature (30 °C) | 0.061 ± 0.002 | 38.6 | <0.001 |
|  | Diet (low) | -0.013 ± 0.002 | -6.9 | <0.001 |
|  | Temperature (25 °C) x Diet (low) | -0.019 ± 0.003 | -7.3 | <0.001 |
|  | Temperature (30 °C) x Diet (low) | -0.029 ± 0.003 | -10.9 | <0.001 |
