# Supplementary material for: Larval diet and temperature alter mosquito immunity and development: using body size and developmental traits to track carry-over effects on longevity
Source: Parasit Vectors. 2023 Nov 22;16:434. doi: 10.1186/s13071-023-06037-z (PMC10666368; doi:10.1186/s13071-023-06037-z)
Supplement: Supplementary file 6 — Additional file 6. Table S6: Linear mixed models of the influence of larval diet and rearing temperature on female Ae. albopictus body size. [file 13071_2023_6037_MOESM6_ESM.docx]

**Table S6.** Linear mixed models of the influence of larval diet and rearing temperature on female *Ae. albopictus* body size.

| **Measure** | **Effect** | **Estimate ± SE** | ***t* value** | **Pr > *z*** |
| --- | --- | --- | --- | --- |
| pupal wet weight | Intercept | 2.521 ± 0.050 | 50.6 | <0.001 |
|  | Temperature (25 °C) | 0.077 ± 0.069 | 1.1 | NS |
|  | Temperature (30 °C) | 0.355 ± 0.069 | 5.2 | <0.001 |
|  | Diet (low) | -0.451 ± 0.076 | -5.9 | <0.001 |
|  | Temperature (25 °C) x Diet (low) | -0.277 ± 0.108 | -2.6 | 0.011 |
|  | Temperature (30 °C) x Diet (low) | -0.696 ± 0.109 | -6.4 | <0.001 |
| wing length | Intercept | 2.592 ± 0.021 | 124.1 | <0.001 |
|  | Temperature (25 °C) | -0.082 ± 0.032 | -2.6 | 0.011 |
|  | Temperature (30 °C) | -0.090 ± 0.032 | -2.8 | 0.006 |
|  | Diet (low) | -0.176 ± 0.033 | -5.3 | <0.001 |
|  | Temperature (25 °C) x Diet (low) | -0.098 ± 0.049 | -2.0 | 0.045 |
|  | Temperature (30 °C) x Diet (low) | -0.251 ± 0.049 | -5.1 | <0.001 |
