# Supplementary material for: Larval diet and temperature alter mosquito immunity and development: using body size and developmental traits to track carry-over effects on longevity
Source: Parasit Vectors. 2023 Nov 22;16:434. doi: 10.1186/s13071-023-06037-z (PMC10666368; doi:10.1186/s13071-023-06037-z)
Supplement: Supplementary file 7 — Additional file 7. Figure S1: Effects of temperature and diet (H, high; L, low) on female Ae. albopictus wing length and pupal wet weight. Mean values of groups sharing the same letter were not significantly different (LMM post hoc Tukey pairwise comparisons, P ≥ 0.05). H, high diet regime; L, low diet regime. [file 13071_2023_6037_MOESM7_ESM.docx]

*
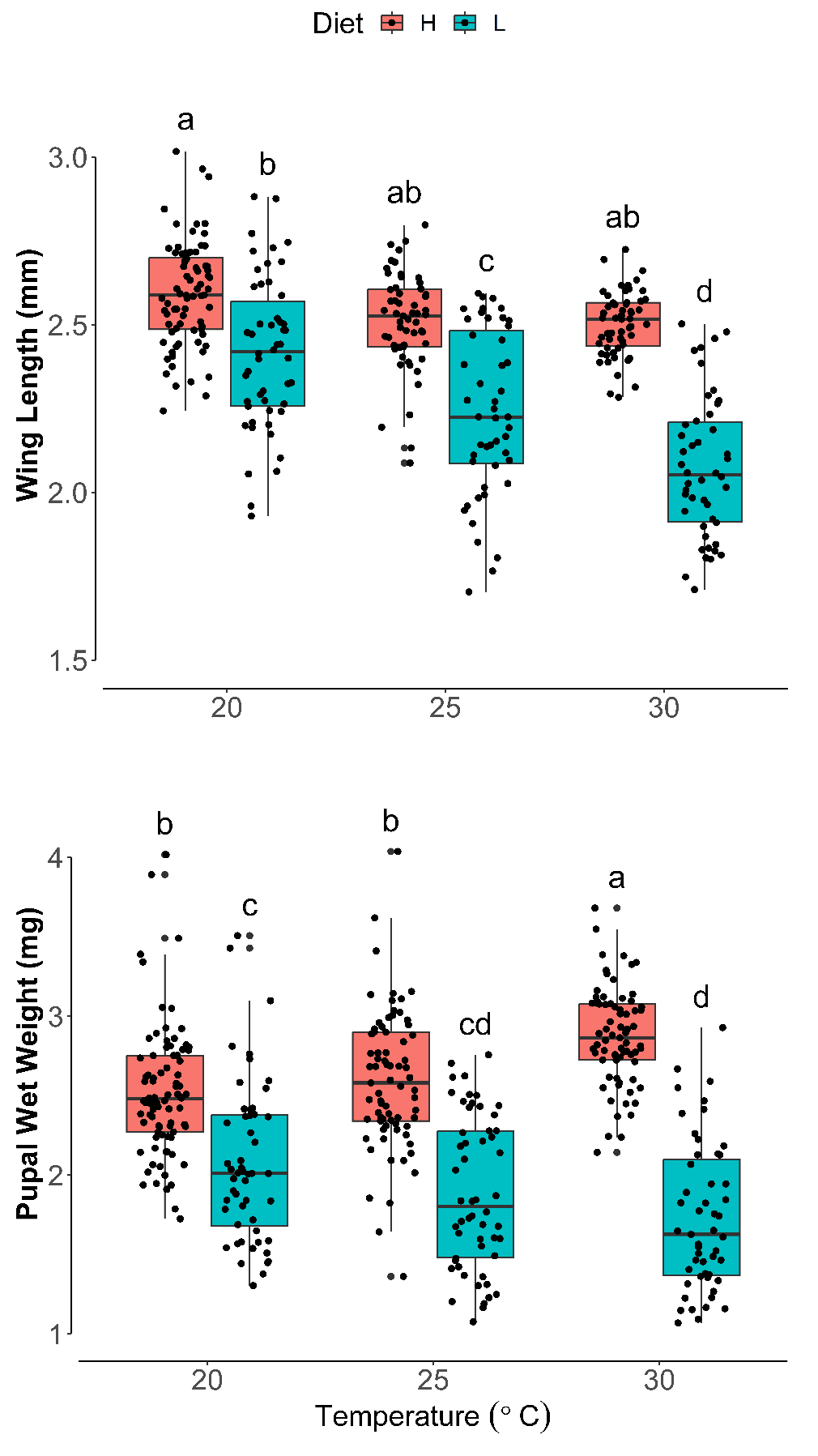
*

**Fig. S1.** Effects of temperature and diet on female *Ae. albopictus* wing length and pupal wet weight. Mean values of groups sharing the same letter were not significantly different (LMM post hoc Tukey pairwise comparisons, P >= 0.05). **H**, high diet regime; **L**, low diet regime
