# Supplementary material for: Larval diet and temperature alter mosquito immunity and development: using body size and developmental traits to track carry-over effects on longevity
Source: Parasit Vectors. 2023 Nov 22;16:434. doi: 10.1186/s13071-023-06037-z (PMC10666368; doi:10.1186/s13071-023-06037-z)
Supplement: Supplementary file 8 — Additional file 8. Figure S2: The relationship between larval development time (L1 to pupation) and adult female longevity for the high and low diets under three different temperatures experienced during the larval stage. H, high diet regime; L, low diet regime. [file 13071_2023_6037_MOESM8_ESM.docx]

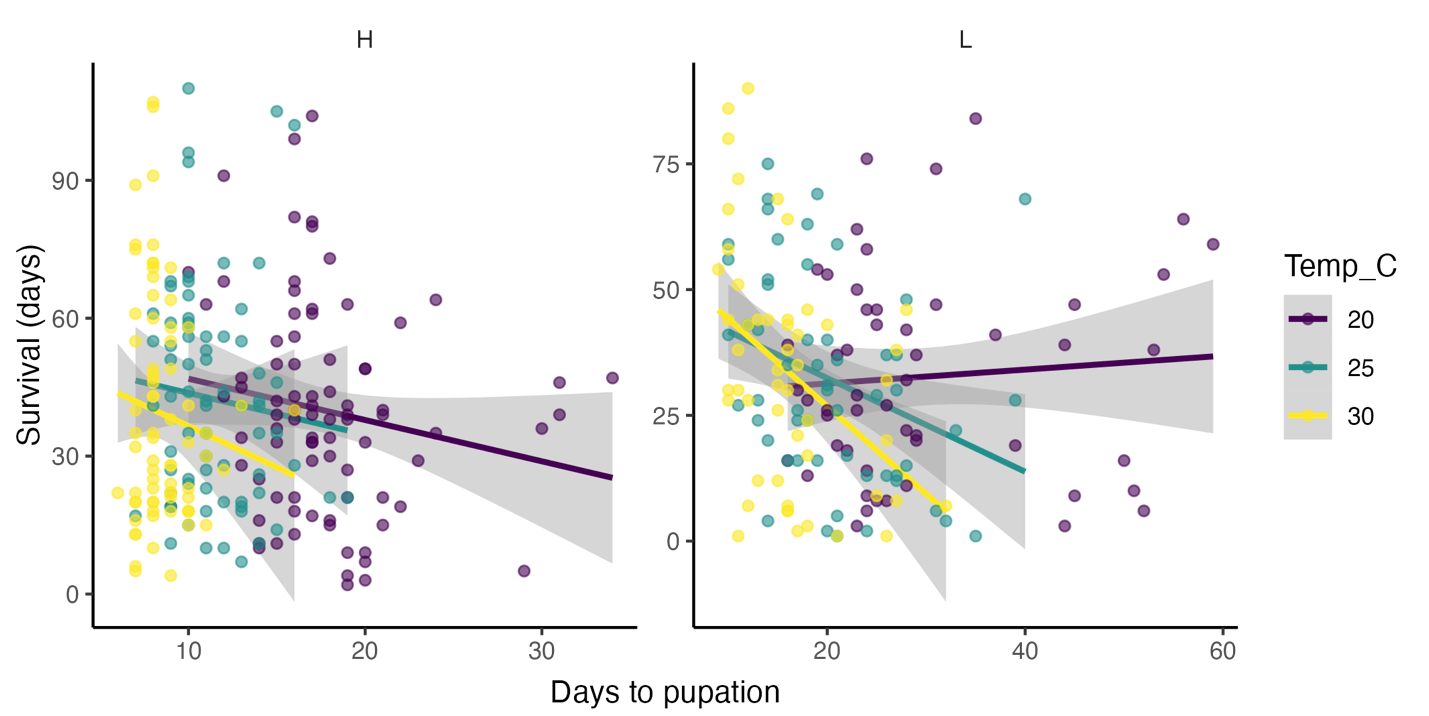


**Fig. S2.** The relationship between larval development time (L1 to pupation) and adult female longevity for the high and low diets under three different temperatures experienced during the larval stage. **H**, high diet regime; **L**, low diet regime
